# Targeted Exon Skipping to Correct Exon Duplications in the Dystrophin Gene

**DOI:** 10.1038/mtna.2014.8

**Published:** 2014-03-18

**Authors:** Kane L Greer, Hanns Lochmüller, Kevin Flanigan, Susan Fletcher, Steve D Wilton

**Affiliations:** 1 Australian Neuromuscular Research Institute, Murdoch University, Perth, Western Australia, Australia; 2 Centre for Neuromuscular & Neurological Disorders, University of Western Australia, Crawley, Western Australia, Australia; 3 Institute of Genetic Medicine, International Centre for Life, University of Newcastle, Newcastle, UK; 4 Nationwide Children's Hospital, Columbus, Ohio, USA; 5 Centre for Comparative Genomics, Murdoch University, Murdoch, Western Australia, Australia

**Keywords:** antisense oligomers, Duchenne muscular dystrophy, duplication mutations, dystrophin, exon skipping

## Abstract

Duchenne muscular dystrophy is a severe muscle-wasting disease caused by mutations in the dystrophin gene that ablate functional protein expression. Although exonic deletions are the most common Duchenne muscular dystrophy lesion, duplications account for 10–15% of reported disease-causing mutations, and exon 2 is the most commonly duplicated exon. Here, we describe the *in vitro* evaluation of phosphorodiamidate morpholino oligomers coupled to a cell-penetrating peptide and 2′-O-methyl phosphorothioate oligonucleotides, using three distinct strategies to reframe the dystrophin transcript in patient cells carrying an exon 2 duplication. Differences in exon-skipping efficiencies *in vitro* were observed between oligomer analogues of the same sequence, with the phosphorodiamidate morpholino oligomer coupled to a cell-penetrating peptide proving the most effective. Differences in exon 2 excision efficiency between normal and exon 2 duplication cells, were apparent, indicating that exon context influences oligomer-induced splice switching. Skipping of a single copy of exon 2 was induced in the cells carrying an exon 2 duplication, the simplest strategy to restore the reading frame and generate a normal dystrophin transcript. In contrast, multiexon skipping of exons 2–7 to generate a Becker muscular dystrophy-like dystrophin transcript was more challenging and could only be induced efficiently with the phosphorodiamidate morpholino oligomer chemistry.

## Introduction

Duchenne muscular dystrophy (DMD) is an X-linked recessive muscle-wasting disease, characterized by progressive weakening of skeletal, respiratory, and cardiac muscle followed by necrosis and fibrosis. DMD affects ~1:3,500 live male births and is associated with delayed motor milestones. Affected individuals commonly lose ambulation before the age of 13 years and most will die in their third decade due to respiratory and cardiac complications.^1,2,3^ DMD occurs as a result of mutations within the *DMD*, located on chromosome Xp21.2, that lead to premature termination of translation. Mutations have been observed across all 79 exons of this 2.4 Mb gene, the most frequent type being exonic deletions and duplications that induce a frame-shift in the protein-coding sequence. Therefore, expression of the 427 kDa muscle-specific dystrophin isoform is ablated, severely compromising the link between the myofiber sarcolemma and cytoskeletal actin, essential for maintaining muscle fiber integrity during contraction and relaxation.^4^

An allelic disorder, Becker muscular dystrophy (BMD), also arises from mutations in the *DMD*, and these are most commonly in-frame deletions that lead to synthesis of an internally deleted dystrophin isoform that retains some function. BMD patients exhibit a later age of onset, slower clinical progression, and a longer lifespan compared to DMD. In some cases the phenotype may be so mild that diagnosis is only made late in life or by chance.^5,6^ The variation in disease progression and severity between DMD and BMD provide the theoretical underpinnings for therapeutic approaches such as antisense oligomer (AO)-induced exon skipping, the goal being to modulate processing of the defective *DMD* transcript to express a BMD-like dystrophin isoform^7,8,9^ that would result in a less severe phenotype.

Duplications account for ~5–10% of all reported mutations in DMD in the Leiden database,^10^ although the incidence has been reported to be higher in other databases and registries.^11^ Regardless of the exact figure, the number of DMD patients with duplications is substantial and demands the development of effective treatment strategies for this class of mutation. Furthermore, restoration of the reading frame for some duplications by excising one copy of a single exon duplication could permit expression of a normal dystrophin transcript. This could potentially allow synthesis of a normal dystrophin isoform, in contrast to restoring the reading frame around the more common deletions where only a BMD-like isoform can be generated.

Induced exon skipping is an intervention during dystrophin pre-mRNA splicing, based on the principle that removing one or more targeted exons can either remove or bypass protein truncating mutations and allow synthesis of internally deleted, but semifunctional BMD-like dystrophin isoforms.^12,13^ Exon skipping can restore the reading frame around dystrophin genomic deletions, the most common type of *DMD* lesion, or induce excision of a single in-frame exon to remove an intraexonic protein truncating mutation.^7,14^ However, the capacity of exon-skipping strategies to correct dystrophin duplications has not been extensively explored. Here, we report studies on targeted excision of dystrophin exon 2, the most commonly duplicated exon in DMD.^10^ By-passing the protein truncating mutation arising from exon 2 duplication can in theory be achieved by applying three distinct strategies: (i) excise only one of the duplicated exons; (ii) remove both duplicated exons and induce the reinitiation of translation in exon 3 or 6,^15,16^ or (iii) remove both duplicated exons, in addition to exons 3, 4, 5, 6, and 7 to restore the reading frame of the modified dystrophin transcript. We show that single exon 2 skipping can be induced in a dose-dependent manner and that the selection of splice switching oligomer chemistry has a major influence on splice-switching efficiency.

## Results

**Figure 1:**
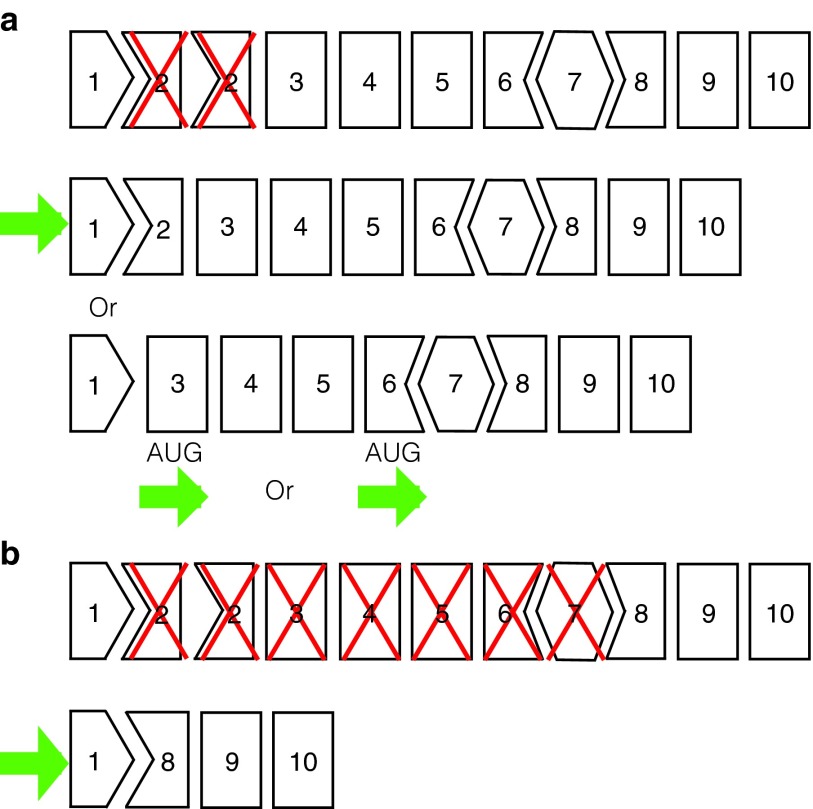
**AO strategies to restore dystrophin expression in the presence of a frame-shifting exon 2 duplication.** (**a**) Possible outcomes resulting from targeting of exon 2 only include single exon skipping (and a resultant in-frame transcript) or excising both copies of exon 2 and relying on reinitiation of translation from exon 3 or 6. (**b**) Induction of multiple exon skipping targeting exons 2–7 to restore the reading frame. AO, antisense oligomer.

The simplest and preferable strategy would be to remove just one copy of the duplicated exon, resulting in a full-length dystrophin transcript that encodes the normal protein. If both copies of exon 2 were removed from the dystrophin mRNA, then it is possible that reinitiation of translation could occur in exons 3 or 6 (**Figure 1a**).^15,16^ Should single exon skipping prove unfeasible, or reinitiation of translation proves inefficient from skipping both exon 2s, the alternate exon-skipping strategy would be to excise exons 2–7 (**Figure 1b**).

**Figure 2:**
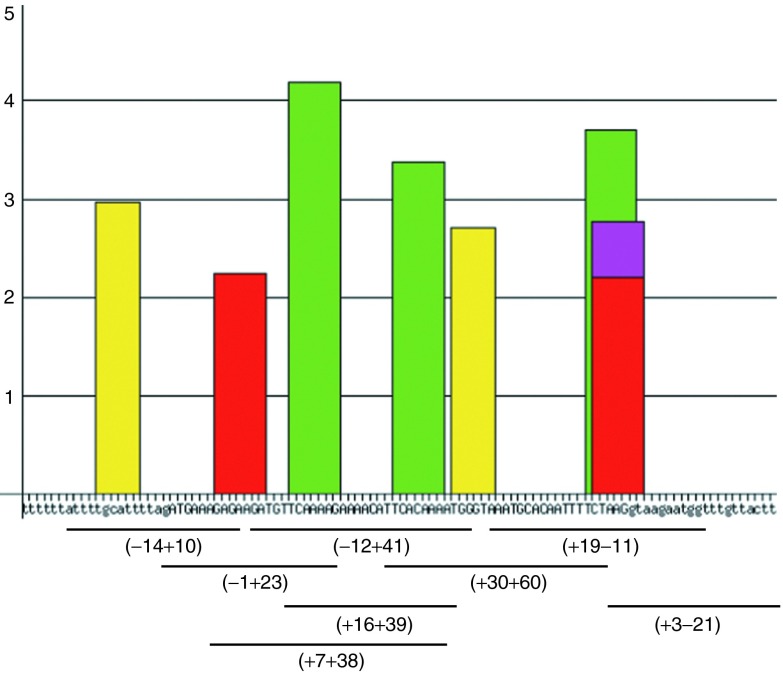
**Schematic showing predicted** **SR proteins binding motifs in dystrophin exon 2, with AO annealing sites indicated.** Lower case letters indicates intronic sequences, while the exon is shown in upper case. The colored bars represent different motifs for predicted SR binding proteins. The heights of the bars indicate predicted binding strength. Red: SRSF1; purple: SRSF1 (IgM-BRCA1); green: SRSF5; yellow: SRSF6. AO, antisense oligomer; SR, specific serine/arginine rich; SRSF, specific serine/arginine rich splicing factor.

**Figure 3:**
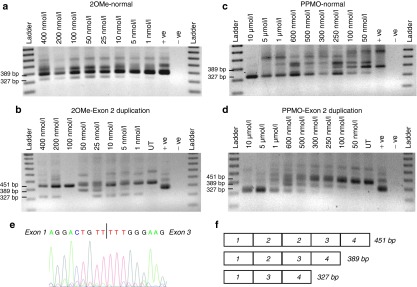
**AO-induced excision of dystrophin exon 2 from normal human myogenic cells and patient dystrophin myogenic cells with a duplication of exon 2, using oligomers of the 2OMe and PPMO chemistries.** Myogenic cells were either transfected with 2OMe AO:cationic lipoplexes at the concentrations indicated and incubated for 24 hours, or transfected with PPMOs and incubated for 96 hours. Total RNA was extracted and nested reverse-transcriptase PCR was undertaken across exons 1–4. The normal, full length, and exon 2-deleted products are 389, 451, and 327 bp, respectively. (**a**) Normal human myogenic cells transfected with 2OMe; (**b**) exon 2 duplication cells with 2OMe; (**c**) normal human myogenic cells with PPMO; (**d**) exon 2 duplication cells with PPMO; (**e**) sequence chromatogram showing exon 1 being spliced to exon 3; (**f**) schematic indicating exonic combinations and predicted amplicon size. AO, antisense oligomer; PPMO, phosphorodiamidate morpholino oligomer, coupled to a cell-penetrating peptide; UT: amplicon from untreated exon 2 duplication cells; +ve: amplicon from untreated normal cells; −ve: no template PCR negative control; 2OMe, 2′O methyl phosphorothioate oligomers.

The coding sequence for exon 2 in the dystrophin mRNA, along with 25 bases of the flanking intronic sequences was interrogated by ESE Finder 3.0 to predict motifs involved in processing the dystrophin pre-mRNA. AOs were designed to anneal to the known splice sites and predicted enhancer motifs across exon 2 and flanking intronic sequences (**Figure 2**). All oligomer sequences (**Table 1**) were first evaluated as 2′O methyl phosphorothioate oligomers (2OMe AO) after transfection into normal human myogenic cells, and only showed modest or no target exon exclusion from the transcript (data images not shown, densitometry results summarized in **Table 1**). One of the more promising compounds, AO H2A(+12+41) induced minimal exon 2 skipping (**Figure 3a**). There were indications of dose-dependent exon skipping at AO transfection concentrations between 1 and 50 nmol/l, but robust exon 2 skipping was not induced, even at the high AO concentrations. Nonoverlapping AOs were combined into cocktails of two or more compounds and transfected into cultured normal cells, but there was no increase in exon-skipping efficiencies relative to that induced by the single compounds (data not shown).

All the 2OMe AOs targeting exon 2 were tested in two unrelated myogenic dystrophic cell strains, DMD 2173 and DMD 2174, both carrying duplications of dystrophin exon 2. Again, H2A(+12+41) was the most effective AO at inducing exon 2 skipping in both cell strains, with induction of dystrophin transcripts missing one or both copies of exon 2, albeit at levels not substantially higher than that induced in the normal cells (**Figure 3b**). Other 2OMe AOs targeting exon 2 did not induce exon skipping or resulted in less than 5% exon 2 skipping in these patient cells, and therefore were excluded from further studies (data not shown). Cocktails containing all possible combinations of two or more nonoverlapping AOs, targeting exon 2, were transfected into both cell strains, but this did not lead to improved skipping compared to a single 2OMe AO (data not shown).

H2A(+12+41) was synthesized as a phosphorodiamidate morpholino oligomer, coupled to a cell-penetrating peptide (PPMO), and transfected into normal human myogenic cells. Robust skipping of exon 2 was achieved, with >80% efficiency at concentrations of 250 nmol/l and above (**Figure 3c**). When transfected into the DMD cell strains, this PPMO induced consistent and robust exon skipping, and dystrophin transcripts missing one or both copies of exon 2 were induced in a dose-dependent manner (**Figure 3d**). Both DMD 2173 and DMD 2174 showed similar effects after PPMO transfection, with one or both of the duplicated exons being removed in a dose-dependent manner, at concentrations similar to that required to excise exon 2 from the normal dystrophin transcript. Exon skipping of only one of the duplicated exons was also induced in a dose-dependent manner and was readily apparent after transfection at PPMO concentrations of 100–600 nmol/l (**Figure 3d**).

**Figure 4:**
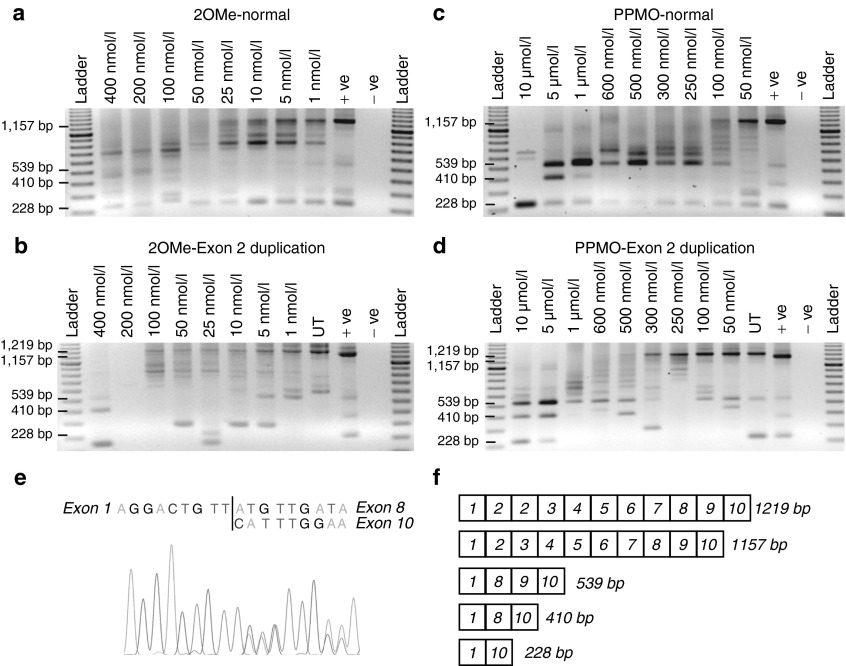
**Induced removal of dystrophin exons 2–7 from normal human myogenic and exon 2 duplication myogenic cells using 2OMe AO and PPMO cocktails.** Primary human and dystrophic myogenic cell cultures were transfected with either 2OMeAO:cationic lipoplexes at the concentrations indicated and incubated for 24 hours or transfected with PPMO conjugates targeting dystrophin exons 2–7 and left for 96 hours. Total RNA was extracted and nested RT-PCR was undertaken across exons 1–10. The normal, full length, and exon 2–7 deletion transcripts are represented by products of 1,157, 1,219, and 539 bp, respectively. (**a**) Normal myogenic cells treated with the 2OMe cocktail. (**b**) Exon 2 duplication cells treated with the 2OMe cocktail. (**c**) Normal myogenic cells treated with the PPMO cocktail. (**d**) Exon 2 duplication cells treated with the PPMO cocktail. (**e**) Sequence chromatogram confirming identity of 539 and 210 bp amplicons. Mixed sequencing peaks are generated downstream of exon 1 due to templates representing transcripts missing exons 2–7 and 2–9. (**f**) Schematic indicating induced exon-skipping product sizes. AO, antisense oligomer; PPMO, phosphorodiamidate morpholino oligomer, coupled to a cell-penetrating peptide; UT: amplicon from untreated exon 2 duplication cells; +ve: amplicon from untreated normal cells; −ve: no template PCR negative control; 2OMe, 2′O methyl phosphorothioate oligomers.

As another splice-switching strategy, we investigated multiple exon skipping to excise exons 2–7 as a single block, the minimal rearrangement necessary to induce an in-frame transcript in lieu of skipping a single copy of exon 2. Exons 2–7 were targeted for removal by AOs (**Table 2**) previously optimized for single exon skipping,^17^ combined in equimolar amounts and transfected into normal- and patient-derived cells. 2OMe AOs transfected as cationic lipoplexes were unable to induce consistent removal of the exon 2–7 block from the full-length transcript, with many intermediate products present and an apparent ablation of the full-length transcript after transfections of 50 nmol/l and above in normal cells (**Figure 4a**), or 200 nmol/l and above in patient-derived cells (**Figure 4a**,**b**).

In contrast, efficient multiple exon skipping was induced using the same oligomer sequence combinations administered as PPMOs. Exons 2–7 were removed efficiently in a dose-dependent manner, with a corresponding depletion of the full-length transcript product in both normal- and patient-derived cells. Ablation of the full-length transcript was complete after transfection of normal cells at total combined oligonucleotide concentrations of 250 nmol/l and above, while higher concentrations were needed to modify the expression of the exon 2 duplicated dystrophin isoform (**Figure 4c**,**d**). A 410 bp amplicon was observed in several lanes and this was found to represent sporadic exon 9 skipping in addition to multiexon skipping of exons 2–7. Sporadic exon 9 skipping has been reported previously and does not disrupt the reading frame.^18^ A smaller amplicon of 228 bp was also observed and found to represent the removal of exons 2–9 a transcript that has also been reported previously, and was detected in untreated normal and patient cells.^19^ The identity of the 539 and 228 bp amplicons can be deduced from the sequence chromatogram shown in **Figure 4e**. Normally, a mixed sequence chromatogram would require purified templates, but in this case, the sequence was unambiguous to the end of exon 1 and then mixed peaks could easily be read to identify the start of exons 8 or 10.

## Discussion

Every dystrophin exon, excluding the first and last can be excised from the mature gene transcript, albeit with considerable variation in the efficiency of exon removal.^17^ Dystrophin exon 2 is relatively small, consisting of only 62 nucleotides and is flanked by introns 1 and 2 of 82,853 and 170,318 bases, respectively. The short length of exon 2 allowed the exon to be covered with a few oligonucleotides. H2A(−14+10), H2A(+12+41), and H2A(+19−11) were designed to target exon 2 with H2A(+12+41) inducing the highest level of exon skipping. Subsequent overlapping AOs were designed based primarily on H2A(+12+41), but no significant improvements in AO efficiency were apparent, as assessed after transfection in normal myogenic cells, and therefore, further studies focused on the H2A(+12+41).

The application of AO-induced exon skipping to correct duplications has to date been minimal, and we are aware of only two studies investigating practical exon skipping to correct duplications in DMD. We have described the correction of an exon 18 duplication by excising both copies of exon 18, as well as exon 17,^14^ whereas Aartsma-Rus *et al.*^7^ have demonstrated successful skipping of duplicated exons 44 and 45, but did not achieve skipping of a larger duplication involving exons 52–62. Here, we sought to address duplications of exon 2, the most frequently duplicated exon in DMD.

Technically, three distinct exon-skipping strategies could be used to induce functional dystrophin expression in cells from patients with duplications of exon 2. The simplest and preferable strategy would be to remove just a single copy of exon 2 from the *DMD* transcript, as this would result in a normal dystrophin transcript and protein. The removal of a single exon 2 from patient cells was achieved by selecting a combination of AO sequence, AO chemistry, and titration of the AO, with PPMOs consistently proving to be more effective than the 2OMe AOs at the removal of exon 2. Direct comparisons between the two different oligomer chemistries are challenging, as the transfection methods vary, but we sought to undertake the 2OMe AO and PPMO evaluations at transfection concentration ranges where dose-dependent exon skipping was evident. The 2OMe AOs are efficiently delivered to cells *in vitro* when complexed to a cationic liposome, but we have found that these lipoplexes can be toxic to the cells at concentrations above 600 nmol/l. PPMOs have a neutral backbone and cannot be complexed with cationic liposomes, but the presence of the cell-penetrating peptide enhances uptake.

We previously reported that AO sequences optimized as 2OMe AO compounds were similarly effective when tested as phosphorodiamidate morpholino oligmers.^20^ However, here, we report substantial differences in *in vitro* exon skipping efficiency between 2OMe AOs, and PPMOs. The PPMO H2A(+12+41) induced very efficient skipping of both copies of exon 2 in normal- and patient-derived cells, suggesting that this compound may be effective in removing a single exon 2 after transfection at lower concentrations. Higher oligomer concentrations removed both copies of exon 2 *in vitro*, and while the resulting dystrophin transcript is out-of-frame, it is possible that dystrophin could still be synthesized through reinitiation of translation in exon 6.^15^ Whether translation reinitiation in exon 6 occurs in these patient cells after removal of both copies of exon 2 is yet to be confirmed. We were unable to demonstrate dystrophin expression by western blotting in treated patient cells where robust skipping of both copies of exon 2 was induced. However, this was not unexpected, since the DMD patient cells used in these experiments were derived from skin fibroblasts and forced into myogenic lineage with a MyoD expressing adenovirus. While the myogenic capacity of such cells is generally sufficient for RNA-based studies, the process is not efficient and detection of dystrophin from normal skin fibroblasts treated in this manner is technically difficult. Nevertheless, protein translation reinitiation has been reported in patients carrying nonsense and frame-shifting mutations in exons 1^15^ and 2.^21^ It is possible that some dystrophin is produced, despite the frame-shifting exclusion of exon 2, but at levels below that detected by our assays.

A technically more challenging alternative strategy to single exon 2 skipping, or dual exon skipping and reinitiation of translation was to remove exons 2–7. Although we have previously achieved skipping of seven consecutive exons (20–26) in the *mdx* mouse^22^ after transfection with 2OMe AOs, we were not able to achieve skipping of the six consecutive exons (2–7) from the human dystrophin transcript with the same oligomer chemistry. We had previously shown that combining individually optimized 2OMe AOs does not necessarily result in the most efficient multiexon skipping cocktails^20^ and we have also observed that some exon combinations are easier to skip than others (unpublished data). However, when the same oligomer sequences, prepared as PPMOs were transfected into normal- and patient-derived cells, robust exon skipping of the target exon block was achieved in a dose-dependent manner. Remarkably, the multiexon skipping appeared very efficient with the PPMO chemistry. Although PPMO concentrations of 250 nmol/l or higher were needed for robust skipping of exons 2–7 in normal cells, this is the combined concentration of the seven oligomers, which are present at individual concentrations of <30 nmol/l, whereas one copy of exon 2 was skipped in 28% of the total transcript products, as assessed by densitometry, after transfection with the single PPMO targeting exon 2 at a concentration of 100 nmol/l in normal cells. Hence, it would appear that multiple exon skipping induced by PPMOs may not require proportionally higher concentrations of oligomers and the indications are that there is some synergy between the oligomers that may weaken selection of the block of targeted exons. Further work is needed to confirm this observation and establish whether the phenomenon is restricted to certain domains of the dystrophin gene transcript. Nevertheless, this study does give some hope that a cocktail of splice-switching oligomers may be a viable option to address clustered DMD-causing mutations.

Evidence suggests that removal of exons 2–7 may result in a functional dystrophin isoform, capable of reducing the severity of DMD. According to Banks *et al.*^23^, loss of the N-terminal domain leads to a mild BMD phenotype and patients with deletions of exons 3–7 commonly present with low-level basal dystrophin and an intermediate phenotype.^19,24,25^ Mutations in this region are described as “leaky”, and patient cells, missing dystrophin exons 3–7 and 5–7, that were treated *in vitro* with AOs targeting exon 8 to restore the reading frame express dystrophin that was readily detectable by western blotting.^26^

In summary, in developing antisense strategies to overcome mutations in the *DMD*, it is evident that the AO chemistry, annealing target and concentration are crucial factors in determining splice-switching efficacy. This is equally relevant not only for patients with exon 2 duplications but also for patients with all dystrophin mutations. Consistent with our previous studies, we find the PPMO chemistry to be better suited for splice-switching oligomers to address duplications and induce multiexon skipping *in vitro*. The current Eteplirsen clinical trials are showing great promise^27^ and these studies indicate the applicability of this oligomer chemistry to other *DMD* lesions, in particular duplications.

## Materials and methods

*AO design and synthesis.* Splice-switching AOs were designed to anneal to splicing motifs at the intron:exon boundaries, as well as exon splice enhancer (ESE) motifs as predicted by the web-based application, ESEfinder.^28^ 2OMe AOs were prepared on an Expedite 8909 Nucleic acid synthesizer (Applied Biosystems) using the 1 μmol/l thioate synthesis protocol. PPMOs were supplied by Sarepta Therapeutics (Cambridge, MA). Oligomer nomenclature is based on that described by Mann *et al.*^8^ and indicates oligomer annealing coordinates.

*Cell propagation.* Normal primary human myoblasts were cultured from surplus biopsy material taken from healthy individuals during elective surgery at Royal Perth Hospital, Perth, Western Australia. Informed consent was obtained and the project was approved by the Human Ethics Committee of the University of Western Australia (approval number, RA4/100/2295). Primary human myogenic cells were prepared as described by Rando and Blau^29^ with minor modifications,^30^ and grown in Hams F10 (Gibco; Life Technologies, Melbourne, Australia) supplemented with fetal bovine serum (Serana, Bunbury, Australia) and chick embryo extract (US Biological, Swampscott, MA). Primary fibroblasts were derived from skin biopsies taken, after informed consent, from DMD patients with frame-shifting duplications of only exon 2 in the dystrophin gene (Newcastle University ethics approval number 08/H0906/28+5).

Prior to transfection, patient fibroblasts were induced in to the myogenic lineage with a MyoD expressing adenovirus, Ad5.f50.AdApt.MyoD (Native Antigen Company, Oxford, UK),^31^ at an multiplicity of infection of 200 and allowed to differentiate in 5% horse serum (Gibco; Life Technologies) in Dulbecco's modified Eagle medium (Life Technologies) for 72 hours. Cells were seeded at 3 × 10^4^ per well in 24-well plates that had been sequentially pretreated for 1 hour with 50 μg/ml poly d-Lysine (Sigma, Sydney, Australia) and 100 μg/ml Matrigel (BD Biosciences, Sydney, Australia).^26^

*Transfection.* Myogenic cells were transfected with either 2OMe AO Lipofectamine 2000 (L2K; Life Technologies) lipoplexes at a 1:1 ratio in Opti-MEM (Life Technologies) according to the manufacturer's instructions, or PPMO in Opti-MEM. The media was replaced after ~24 hours for 2OMe AO transfections, or after 96 hours for PPMO transfections.

*RNA extraction and RT-PCR assays.* Trizol (Life Technologies) was used to extract total RNA from cultured cells, according to the manufacturer's guidelines. RT-PCR primers were designed to amplify several exons either side of the target exons, in order to minimize preferential amplification of shortened (skipped) transcript products. This assay also allows detection of other RNA processing events, including cryptic splice site activation or removal of nontargeted exons. Primer sequences for cDNA synthesis and amplification are shown in **Table 3**.

RT-PCR was performed on 50 ng of total RNA using a Superscript III One-Step RT-PCR System with Platinum Taq DNA Polymerase (Life Technologies) as described by Wilton *et al.*^17^ After 35 cycles, a 1-µl aliquot was removed and used as template in a nested PCR for 30 cycles using AmpliTaq Gold (Applied Biosystems, Melbourne, Australia).

*Gel analysis and Imaging.* RT-PCR products were resolved on a 2% agarose gel in Tris-acetate EDTA buffer using a 100 bp DNA ladder (Life Technologies) as size standards. Exon-skipping efficiency was estimated by analyzing densitometry images captured by a Chemi-Smart 3000 system (Vilber Lourmat) using Chemi-Capt software for image acquisition and Bio-1D software for analysis, as described previously.^20^ All transfections and RT-PCR analyses were carried out in duplicate.

## Figures and Tables

**Table 1:**
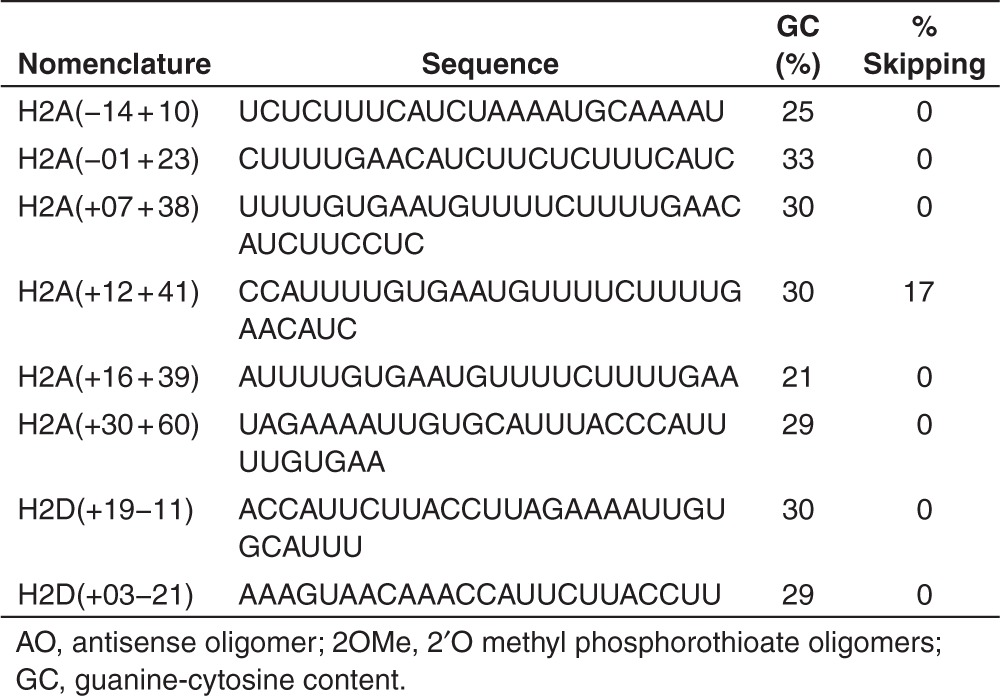
Nucleotide sequences, annealing coordinates, GC content, and exon-skipping efficiency (percentage of shortened product relative to full-length amplicon) of 2OMe AOs designed to remove dystrophin exon 2 after transfection at 50 nmol/l

**Table 2:**
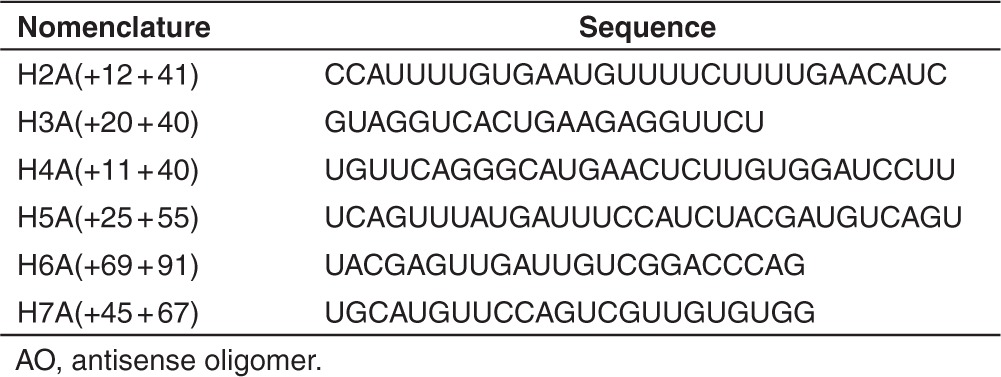
Nucleotide sequences and annealing coordinates of AOs designed to remove dystrophin exons 2–7

**Table 3:**
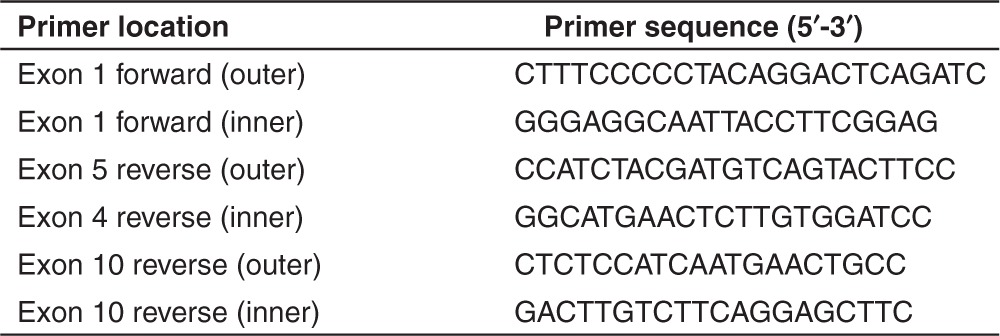
Primer sequences for PCR
